# Supplementary material for: PD-1/LAG-3 co-signaling profiling uncovers CBL ubiquitin ligases as key immunotherapy targets
Source: EMBO Mol Med. 2024 Jul 19;16(8):2. doi: 10.1038/s44321-024-00098-y (PMC11319776; doi:10.1038/s44321-024-00098-y)
Supplement: Supplementary file 1 — Appendix [file 44321_2024_98_MOESM1_ESM.pdf]

Appendix.

Appendix Table S1. Exact p values for all figures.

Figure 1a 2
Figure 2d 2
Figure 2e 3
Figure 2f 4
Figure 3b 4
Figure 3c 5
Figure 3d 5
Figure 3g 5
Figure 3h 5
Figure 4a 6
Figure 5b 6
Figure 5c 6
Figure 5d 6
Figure 5e 7
Figure 6a 7
Figure 6b and 6c 8
Figure 6e 8
Figure 6f 8
Figure 7b 9
Figure 7c and 7d 9
Figure EV2a 9
Figure EV2b 10
Figure EV2c 10
Figure EV3d 11
Figure EV3e 11

Figure 1a

Figure 1a		
TCGA cancer (n=12159)	adj.p	(-Log10 adj.p)
ACC (n=79)	8.31E-06	5.08055927
BLCA (n=408)	3.65E-90	89.43800116
BRCA (n=1100)	8.30E-163	162.0806629
BRCA-Basal (n=191)	4.44E-42	41.35237834
BRCA-Her2 (n=82)	1.29E-11	10.88883716
BRCA-LumA (n=568)	4.29E-68	67.36723802
BRCA-LumB (n=219)	3.04E-27	26.51781431
CESC (n=306)	1.26E-74	73.89890635
CHOL (n=36)	0.000545683	3.263059631
COAD (n=458)	3.04E-103	102.5164187
DLBC (n=48)	0.016769561	1.775478302
ESCA (n=185)	1.67E-46	45.77750544
GBM (n=153)	2.98E-09	8.52536619
HNSC (n=522)	1.96E-166	165.7078207
HNSC-HPV- (n=422)	3.13E-130	129.5049439
HNSC-HPV+ (n=98)	4.13E-32	31.38368297
KICH (n=66)	5.73E-14	13.2419523
KIRC (n=533)	1.61E-155	154.7927631
KIRP (n=290)	1.32E-39	38.87849622
LGG (n=516)	9.34E-23	22.02980828
LIHC (n=371)	2.06E-43	42.6871728
LUAD (n=515)	7.32E-82	81.13549472
LUSC (n=501)	9.32E-128	127.0303523
MESO (n=87)	1.44E-06	5.840652333
OV (n=303)	4.18E-23	22.37865511
PAAD (n=179)	1.13E-21	20.94512233
PCPG (n=181)	2.04E-09	8.690225772
PRAD (n=498)	2.59E-31	30.5873415
READ (n=166)	2.12E-30	29.6727445
SARC (n=260)	6.44E-26	25.19087787
SKCM (n=471)	1.07E-193	192.9719663
SKCM-Metastasis (n=368)	2.73E-140	139.5642378
SKCM-Primary (n=103)	1.77E-42	41.75199477
STAD (n=415)	1.12E-76	75.94974392
TGCT (n=150)	9.71E-39	38.01275453
THCA (n=509)	7.76E-86	85.10999057
THYM (n=120)	0.052461039	1.280163112
UCEC (n=545)	7.62E-44	43.1178219
UCS (n=57)	0.000126354	3.898410648
UVM (n=80)	7.56E-26	25.12136457

Figure 2d

Figure 2d		
2way ANOVA (Tukey's multiple comparisons test) day 8	adj p-value	Summary
SC3 vs. PD-1	0.0035	**
SC3 vs. LAG-3	0.0056	**
SC3 vs. PD-1+LAG-3	0.0026	**

Figure 2e

Figure 2e		
**CD3+ (%)**		
**2way ANOVA (Tukey's multiple comparisons test)**	**adj p-value**	**Summary**
SC3 vs. PD-1	<0,0001	****
SC3 vs. LAG-3	<0,0001	****
SC3 vs. PD-1+LAG-3	<0,0001	****
**CD3+Ki67+ (%)**		
**2way ANOVA (Tukey's multiple comparisons test)**	**adj p-value**	**Summary**
SC3 vs. PD-1	<0,0001	****
SC3 vs. LAG-3	<0,0001	****
SC3 vs. PD-1+LAG-3	<0,0001	****
**CD3+ZAP70+ (%)**		
**2way ANOVA (Tukey's multiple comparisons test)**	**adj p-value**	**Summary**
SC3 vs. PD-1	<0,0001	****
SC3 vs. LAG-3	<0,0001	****
SC3 vs. PD-1+LAG-3	<0,0001	****
**CD3+CD4+H2AX+ (%)**		
**2way ANOVA (Tukey's multiple comparisons test)**	**adj p-value**	**Summary**
SC3 vs. PD-1	<0,0001	****
SC3 vs. LAG-3	0.0055	**
SC3 vs. PD-1+LAG-3	0.1759	ns

Figure 2f

Figure 2f		
IL-12/IL-23 p40+ (%)		
2way ANOVA (Tukey's multiple comparisons test)	Adjusted P Value	Summary
SC3 vs. PD-1	0.0318	*
SC3 vs. LAG-3	0.0047	**
SC3 vs. PD-1+LAG-3	0.0268	*
IFNg+ (%)		
2way ANOVA (Tukey's multiple comparisons test)	Adjusted P Value	Summary
SC3 vs. PD-1	0.1482	ns
SC3 vs. LAG-3	0.0036	**
SC3 vs. PD-1+LAG-3	0.0269	*
IFNa/b+ (%)		
2way ANOVA (Tukey's multiple comparisons test)	Adjusted P Value	Summary
SC3 vs. PD-1	0.0329	*
SC3 vs. LAG-3	0.003	**
SC3 vs. PD-1+LAG-3	0.0289	*
IL2+ (%)		
2way ANOVA (Tukey's multiple comparisons test)	Adjusted P Value	Summary
SC3 vs. PD-1	0.0015	**
SC3 vs. LAG-3	0.0011	**
SC3 vs. PD-1+LAG-3	0.0023	**
IL4+ (%)		
2way ANOVA (Tukey's multiple comparisons test)	Adjusted P Value	Summary
SC3 vs. PD-1	0.0033	**
SC3 vs. LAG-3	0.0017	**
SC3 vs. PD-1+LAG-3	0.0064	**
IL17A+ (%)		
2way ANOVA (Tukey's multiple comparisons test)	Adjusted P Value	Summary
SC3 vs. PD-1	0.0054	**
SC3 vs. LAG-3	0.0012	**
SC3 vs. PD-1+LAG-3	0.0052	**

Figure 3b

Figure 3b	
Canonical pathway	(-Log10 p-value)
Nonsense-Mediated Decay (NMD)	36.3
Processing of Capped Intron-Containing Pre-mRNA	36.3
Eukaryotic Translation Initiation	32.8
Eukaryotic Translation Elongation	31.7
Eukaryotic Translation Termination	30.6
SRP-dependent cotranslational protein targeting to membrane	30.4
Response of EIF2AK4 (GCN2) to amino acid deficiency	28.8
Selenoamino acid metabolism	26.9
EIF2 Signaling	25.6
Major pathway of rRNA processing in the nucleolus and cytosol	25.2

Figure 3c

Figure 3c	
**Molecular and cellular functions**	**p-value range**
RNA post-transcriptional modification	1,14E-03 - 1,5E-38
Cellular function and maintenance	1,66E-03 - 8,05E-36
Cell death and survival	1,70E-03 - 3,01E-31
Protein synthesis	7,11E-04 - 1,54E-21
Post-translational modification	5,62E-04 - 4,65E-20

Figure 3d

Figure 3d	
**Upstream regulator**	**(-Log10 p-value)**
RICTOR	36.70553377
MYC	35.06600684
TP53	33.09854168
LARP1	31
HNF4A	29.40011693

Figure 3g

Figure 3g	
**Upstream regulators**	**(-Log10 p-value)**
**LARP1**	12.69036983
**MYC**	7.777283529
**YAP1**	7.415668776
**MLXIPL**	7.257274869
**MYCN**	6.360513511
**Canonical pathways**	**(-Log10 p-value)**
**Major pathway of rRNA processing in the nucleolus and cytosol**	13.1
**Eukaryotic Translation Elongation**	12
**Nonsense-Mediated Decay (NMD)**	11.3
**Eukaryotic Translation Termination**	10.2
**Response of EIF2AK4 (GCN2) to amino acid deficiency**	9.9

Figure 3h

Figure 3h		
**Ordinary one-way ANOVA (Tukey's multiple comparisons test)**	**adj p-value**	**Summary**
Basal Non-responder vs Responder	0.105	ns
Basal Non-responder vs Healthy	0.0917	ns
Postac Non-responder vs Responder	0.0359	*
Postac Non-responder vs Healthy	0.0243	*

Figure 4a

Figure 4a			
	(-Log10 p-value)		
Canonical pathways / Upstream regulators	PD-1	LAG-3	PD-1+LAG-3
EIF2 Signaling	9.610216922	10.83869537	15.26758794
Eukaryotic Translation Initiation	9.423626699	14.6547513	18.06986847
Nonsense-Mediated Decay (NMD)	10.79446003	14.91490246	19.73198276
SRP-dependent cotranslational protein targeting to membrane	8.636244349	15.02240187	18.70706113
Eukaryotic Translation Elongation	10.81617064	16.22626715	20.82119388
TCR	1.967055294	1.998772682	8.526915903
LH	8.905337253	5.129767238	9.976610949
NFkB (complex)	1.606562614	1.844540156	0.953352006
RCN3	3.920453954	8.336101517	10.68536053
IL4	3.528593945	1.651911431	4.194196999

Figure 5b

Figure 5b		
Mann-Whitney test (unpaired, nonparametric, two-tailed)	p-value	Summary
CD8 Healthy vs CD8 patients	0.0062	**
CD4 Healthy vs CD4 patients	0.0167	*

Figure 5c

Figure 5c		
MFI of CBL-B in NSCLC CD4 T cells		
2way ANOVA (Tukey's multiple comparisons test)	adj p-value	Summary
Control vs. α-PD-1	0.0003	***
Control vs. α-LAG-3	0.9851	ns
Control vs. α-PD-1+α-LAG-3	0.0006	***
Control vs. Bispecific	<0,0001	****
MFI of CBL-B in NSCLC CD8 T cells		
2way ANOVA (Tukey's multiple comparisons test)	adj p-value	Summary
Control vs. α-PD-1	0.006	**
Control vs. α-LAG-3	>0,9999	ns
Control vs. α-PD-1+α-LAG-3	0.0062	**
Control vs. Bispecific	0.0005	***

Figure 5d

Figure 5d		
MFI of C-CBL in NSCLC CD4 T cells		
2way ANOVA (Tukey's multiple comparisons test)	adj p-value	Summary
Control vs. α-PD-1	0.9893	ns
Control vs. α-LAG-3	0.9849	ns
Control vs. α-PD-1+α-LAG-3	0.9906	ns
Control vs. Bispecific	<0,0001	****
MFI of C-CBL in NSCLC CD8 T cells		
2way ANOVA (Tukey's multiple comparisons test)	adj p-value	Summary
Control vs. α-PD-1	>0,9999	ns
Control vs. α-LAG-3	0.9403	ns
Control vs. α-PD-1+α-LAG-3	0.999	ns
Control vs. Bispecific	<0,0001	****

Figure 5e

Figure 5e		
Ki67+ proliferating CD4 T cells (%)		
2way ANOVA (Tukey's multiple comparisons test)	adj p-value	Summary
Control vs. α-PD-1	0.9195	ns
Control vs. α-LAG-3	0.9486	ns
Control vs. α-PD-1+α-LAG-3	0.0094	**
Control vs. Bispecific	<0,0001	****
Ki67+ proliferating CD8 T cells (%)		
2way ANOVA (Tukey's multiple comparisons test)	adj p-value	Summary
Control vs. α-PD-1	0.9911	ns
Control vs. α-LAG-3	0.9642	ns
Control vs. α-PD-1+α-LAG-3	0.0037	**
Control vs. Bispecific	<0,0001	****

Figure 6a

Figure 6a		
2way ANOVA (Tukey's multiple comparisons test)	adj p-value	Summary
Control vs. DMSO	>0,9999	ns
Control vs. 500nM	0.9998	ns
Control vs. 750nM	0.9972	ns
Control vs. 1μM	0.7822	ns
Control vs. 2.5μM	0.6211	ns
Control vs. 5μM	0.9059	ns
Control vs. 10μM	0.9945	ns
DMSO vs. 500nM	>0,9999	ns
DMSO vs. 750nM	0.9989	ns
DMSO vs. 1μM	0.7354	ns
DMSO vs. 2.5μM	0.5698	ns
DMSO vs. 5μM	0.8733	ns
DMSO vs. 10μM	0.9892	ns
500nM vs. 750nM	>0,9999	ns
500nM vs. 1μM	0.5311	ns
500nM vs. 2.5μM	0.3767	ns
500nM vs. 5μM	0.6968	ns
500nM vs. 10μM	0.9281	ns
750nM vs. 1μM	0.4175	ns
750nM vs. 2.5μM	0.2839	ns
750nM vs. 5μM	0.5767	ns
750nM vs. 10μM	0.8532	ns
1μM vs. 2.5μM	>0,9999	ns
1μM vs. 5μM	>0,9999	ns
1μM vs. 10μM	0.9903	ns
2.5μM vs. 5μM	0.9987	ns
2.5μM vs. 10μM	0.9491	ns
5μM vs. 10μM	0.9994	ns

Figure 6b and 6c

Figure 6b and 6c		
2way ANOVA (Tukey's multiple comparisons test) day 10	Adj p-value	Summary
Saline vs. aPD1+aLAG3+CBL-Bi	0.0037	**
aLAG-3 vs. aPD1+aLAG3+CBL-Bi	0.0134	*
aPD1+aLAG3 vs. aPD1+aLAG3+CBL-Bi	0.044	*

Figure 6e

Figure 6e		
2way ANOVA (Tukey's multiple comparisons test) day 14	Adj p-value	Summary
Saline vs. aPD1+aLAG3+CBL-bi 10mg/kg	0.0209	*
Saline vs. aPD1+aLAG3+CBL-bi 20mg/kg	0.0098	**
Saline vs. aPD1+aLAG3+CBL-bi 30mg/kg	0.0098	**

Figure 6f

Figure 6f	
Log-rank (Mantel-Cox) test	
P value	<0,0001
P value summary	****
Are the survival curves sig different?	Yes
Log-rank (Mantel-Cox) test aPD-1+aLAG-3 vs aPD1+aLAG3+CBL-bi 10mg/kg	
P value	0.016
P value summary	*
Are the survival curves sig different?	Yes
Log-rank (Mantel-Cox) test aPD-1+aLAG-3 vs aPD1+aLAG3+CBL-bi 20mg/kg	
P value	0.016
P value summary	*
Are the survival curves sig different?	Yes
Log-rank (Mantel-Cox) test aPD-1+aLAG-3 vs aPD1+aLAG3+CBL-bi 30mg/kg	
P value	0.0022
P value summary	**
Are the survival curves sig different?	Yes

Figure 7b

Figure 7b	
**Log-rank (Mantel-Cox) test**	
P value	<0,0001
P value summary	****
Are the survival curves sig different?	Yes
**Log-rank (Mantel-Cox) test αPD-1 + αLAG-3 vs. αPD-1 + αLAG-3 + CBL-Bi**	
P value	0.0012
P value summary	**
Are the survival curves sig different?	Yes
**Log-rank (Mantel-Cox) test αPD-1 + αLAG-3 vs. αPD-1 + αLAG-3 + CBL-Bi + αCD8**	
P value	0.019
P value summary	*
Are the survival curves sig different?	Yes
**Log-rank (Mantel-Cox) test αPD-1 + αLAG-3 + CBL-Bi vs. αPD-1 + αLAG-3 + CBL-Bi + αCD8**	
P value	0.0009
P value summary	***
Are the survival curves sig different?	Yes

Figure 7c and 7d

Figure 7c and 7d		
**2way ANOVA (Tukey's multiple comparisons test)**	**Adj p-value**	**Summary**
αPD-1 + αLAG-3 vs. αPD-1 + αLAG-3 + CBL-Bi	0.0004	***
αPD-1 + αLAG-3 vs. αPD-1 + αLAG-3 + CBL-Bi + αCD8	0.0021	**
αPD-1 + αLAG-3 + CBL-Bi vs. αPD-1 + αLAG-3 + CBL-Bi + αCD8	<0,0001	****

Figure EV2a

Figure EV2a			
**Canonical Pathways**	**PD-1**	**LAG-3**	**PD-1+LAG-3**
T Cell Exhaustion Signaling Pathway	1.629657653	1.629657653	3.26006396
Costimulation by the CD28 family	2.49114611	0	2.19080853
PD-1, PD-L1 cancer immunotherapy pathway	2.353856935	0	2.05378042
MHC class II antigen presentation	0	2.282870167	1.98296479
D-myo-inositol (1,4,5,6)-Tetrakisphosphate Biosynthesis	2.123169324	0	1.82376872
**Upstream regulators**	**PD-1**	**LAG-3**	**PD-1+LAG-3**
HLA-A	3.300879637	3.300879637	6.63650467
PTPN22	2.923461295	2.923461295	5.86114632
SELPLG	2.781354534	2.781354534	5.57291152
KDR	2.742725131	2.742725131	5.49477358
JAK3	2.707252813	2.707252813	5.42308909

Figure EV2b

Figure EV2b		
**Condition**	**Molecular and cellular functions**	**p-value range**
PD-1	Cellular development	6,87E-03 - 4,14E-05
	Cellular function and maintenance	5,34E-03 - 4,14E-05
	Cellular growth and proliferation	6,87E-03 - 4,14E-05
	Cell-to-cell signaling and interaction	8,07E-03 - 8,28E-05
	Cellular compromise	4,92E-03 - 1,24-E-04
LAG-3	Cellular compromise	3,72E-04 - 1,24E-04
	Cell-to-cell signaling and interaction	2,47E-02 - 1,66E-04
	Cellular development	8,65E-03 - 3,31E-04
	Cellular growth and proliferation	8,65E-03 - 3,31E-04
	Cell morphology	1,90-E03 - 3,72E-04
PD-1+LAG-3	Cellular compromise	2,89E-03 - 1,03E-08
	Cellular movement	1,41E-03 - 2,26E-07
	Cell-to-cell signaling and interaction	4,46E-03 - 6,27E-06
	Cellular growth and proliferation	4,46E-03 - 8,75E-06
	Cellular development	4,46E-03 - 7,44E-05

Figure EV2c

Figure EV2c		
**Condition**	**Diseases and disorders**	**p-value range**
PD-1	Cancer	1,28E-02 - 4,14E-05
	Cardiovascular disease	5,34E-03 - 4,14E-05
	Connective tissue disorders	1,08E-03 - 4,14E-05
	Dermatological diseases and conditions	6,87E-03 - 4,14E-05
	Endocrine system disorders	6,41E-03 - 4,14E-05
LAG-3	Cancer	2,95E-02 - 8,28E-05
	Dermatological diseases and conditions	2,19E-03 - 8,28E-05
	Gastrointestinal disease	2,77E-03 - 8,28E-05
	Organismal injury and abnormalities	2,95E-02 - 8,28E-05
	Tumor morphology	3,81E-03 - 8,28E-05
PD-1+LAG-3	Cancer	5,26E-03 - 3,42E-09
	Dermatological diseases and conditions	3,80E-03 - 3,42E-09
	Gastrointestinal disease	5,04E-03 - 3,42E-09
	Organismal injury and abnormalities	5,26E-03 - 3,42E-09
	Tumor morphology	5,12E-03 - 3,42E-09

Figure EV3d

Figure EV3d		
**Condition**	**Molecular and cellular functions**	**p-value range**
**PD-1**	Cellular function and maintenance	1,02E-02 - 1,92E-11
	RNA post-transcriptional modification	4,61E-03 - 3,90E-11
	Cell death and survival	1,02E-02 - 3,08E-10
	Cell morphology	1,02E-02 - 6,06E-09
	Protein synthesis	1,01E-03 - 6,72E-07
**LAG-3**	RNA post-transcriptional modification	1,02E-02 - 1,26E-09
	Cellular function and maintenance	1,98E-02 - 9,65E-09
	Protein synthesis	1,39E-02 - 7,13E-08
	Cell death and survival	2,04E-02 - 1,44E-07
	DNA replication, recombination and repair	2,04E-02 - 2,41E-06
**PD-1+LAG-3**	Cell death and survival	5,33E-03 - 5,86E-15
	RNA post-transcriptional modification	2,96E-04 - 1,84E-14
	Cellular function and maintenance	5,78E-03 -2,75E-14
	Cell-to-cell signaling and interaction	5,86E-03 - 4,97E-09
	Cellular development	5,78E-03 - 5,49E-09

Figure EV3e

Figure EV3e		
**Condition**	**Diseases and disorders**	**p-value range**
**PD-1**	Cancer	1,02E-02 - 1,27E-14
	Organismal injury and abnormalities	1,02E-02 - 1,27E-14
	Infectious diseases	1,02E-02 - 8,72E-11
	Endocrine system disorders	1,02E-02 - 8,12E-09
	Tumor morphology	1,02E-02 - 1,55E-07
**LAG-3**	Cancer	2,04E-02 - 2,00E-08
	Organismal injury and abnormalities	2,04E-02 - 2,00E-08
	Endocrine system disorders	1,77E-02 - 4,23E-08
	Tumor morphology	1,53E-02 - 6,66E-06
	Gastrointestinal disease	2,04E-02 - 6,91E-06
**PD-1+LAG-3**	Cancer	5,86E-03 - 5,11E-25
	Organismal injury and abnormalities	5,86E-03 - 5,11E-25
	Endocrine system disorders	5,86E-03 - 6,43E-15
	Infectious diseases	5,35E-03 - 1,65E-13
	Gastrointestinal disease	5,86E-03 - 1,36E-09
