# Supplementary figures and images for: PD-1/LAG-3 co-signaling profiling uncovers CBL ubiquitin ligases as key immunotherapy targets
Source: EMBO Mol Med. 2024 Jul 19;16(8):2. doi: 10.1038/s44321-024-00098-y (PMC11319776; doi:10.1038/s44321-024-00098-y)

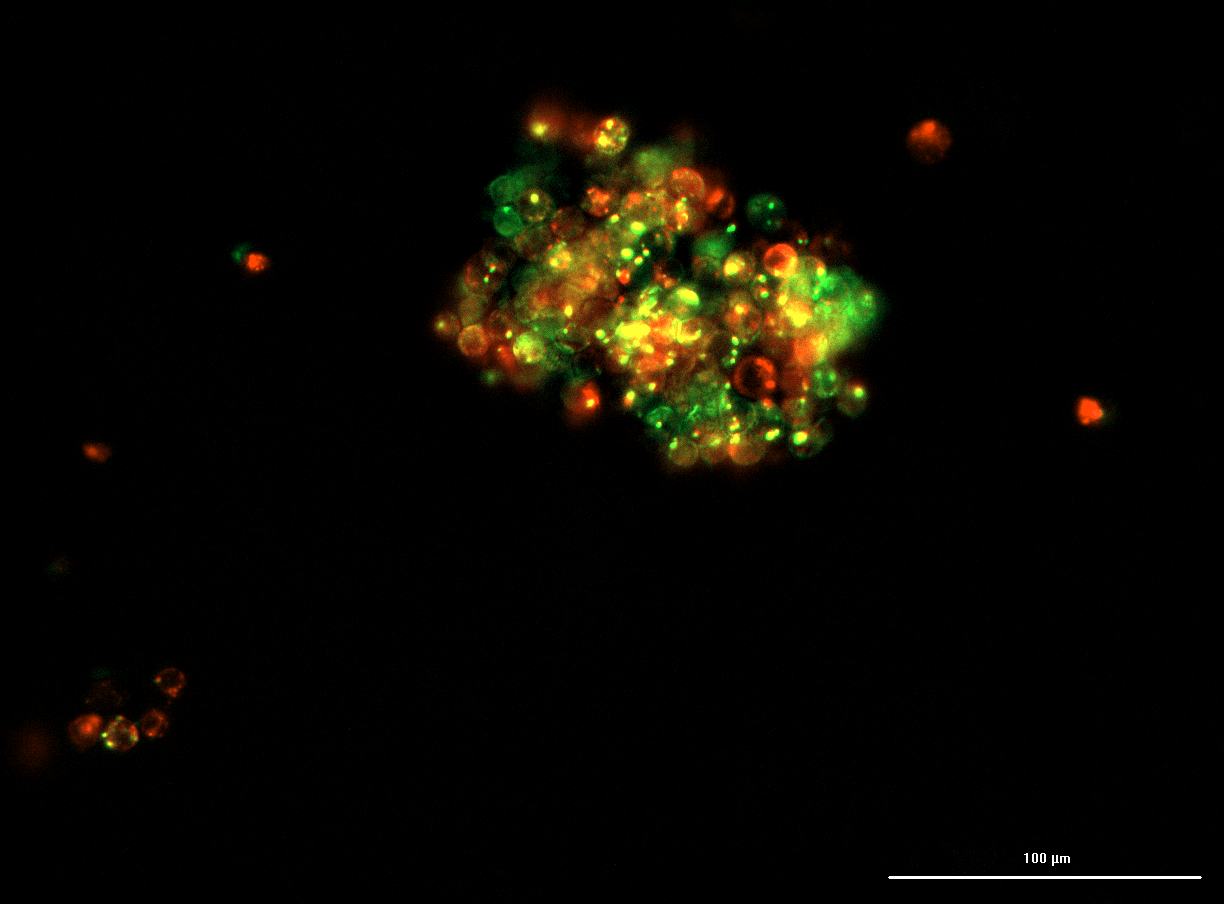

Supplement: Supplementary file 7 — Source data Fig. 2 [file 44321_2024_98_MOESM7_ESM.zip › Fig2/Fig2b/PD1+LAG3.png]

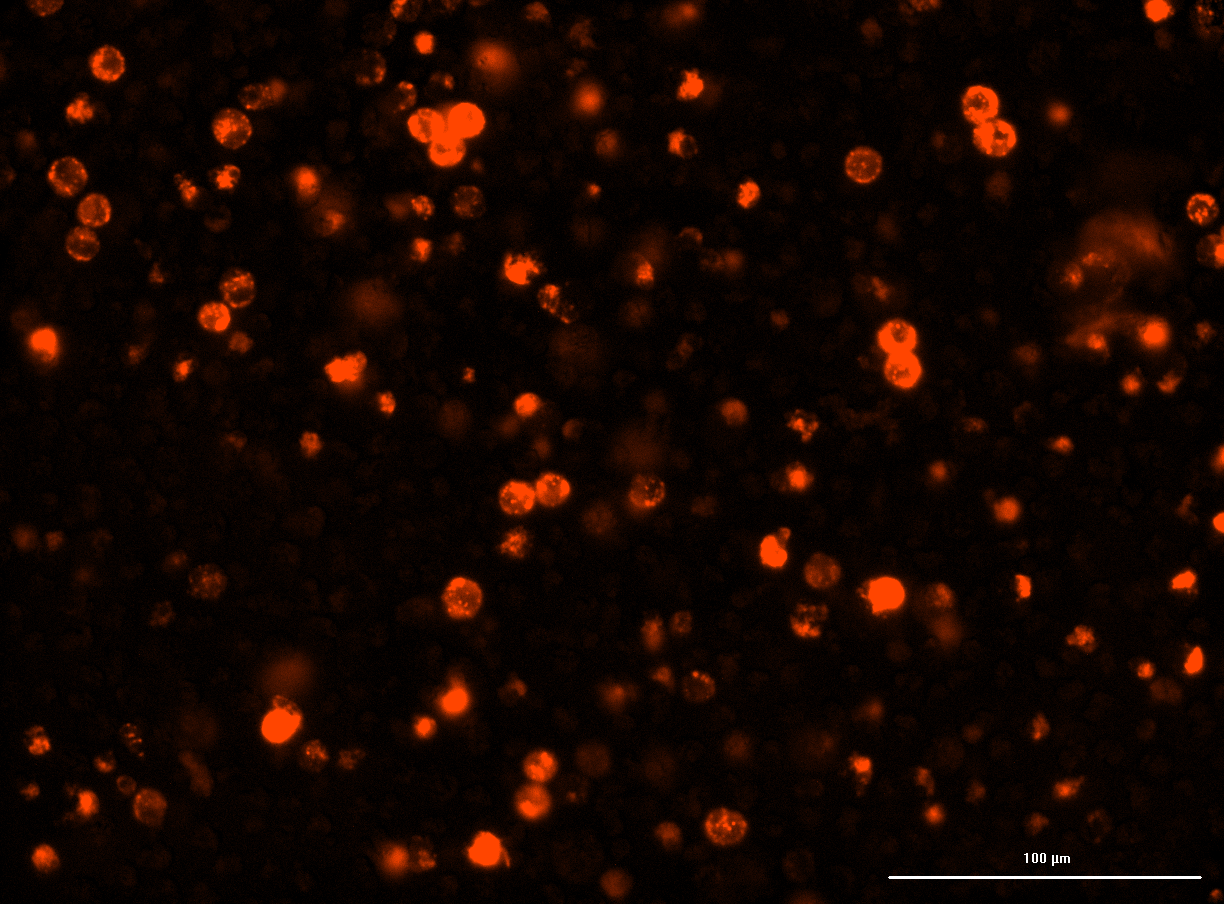

Supplement: Supplementary file 7 — Source data Fig. 2 [file 44321_2024_98_MOESM7_ESM.zip › Fig2/Fig2b/LAG-3.png]

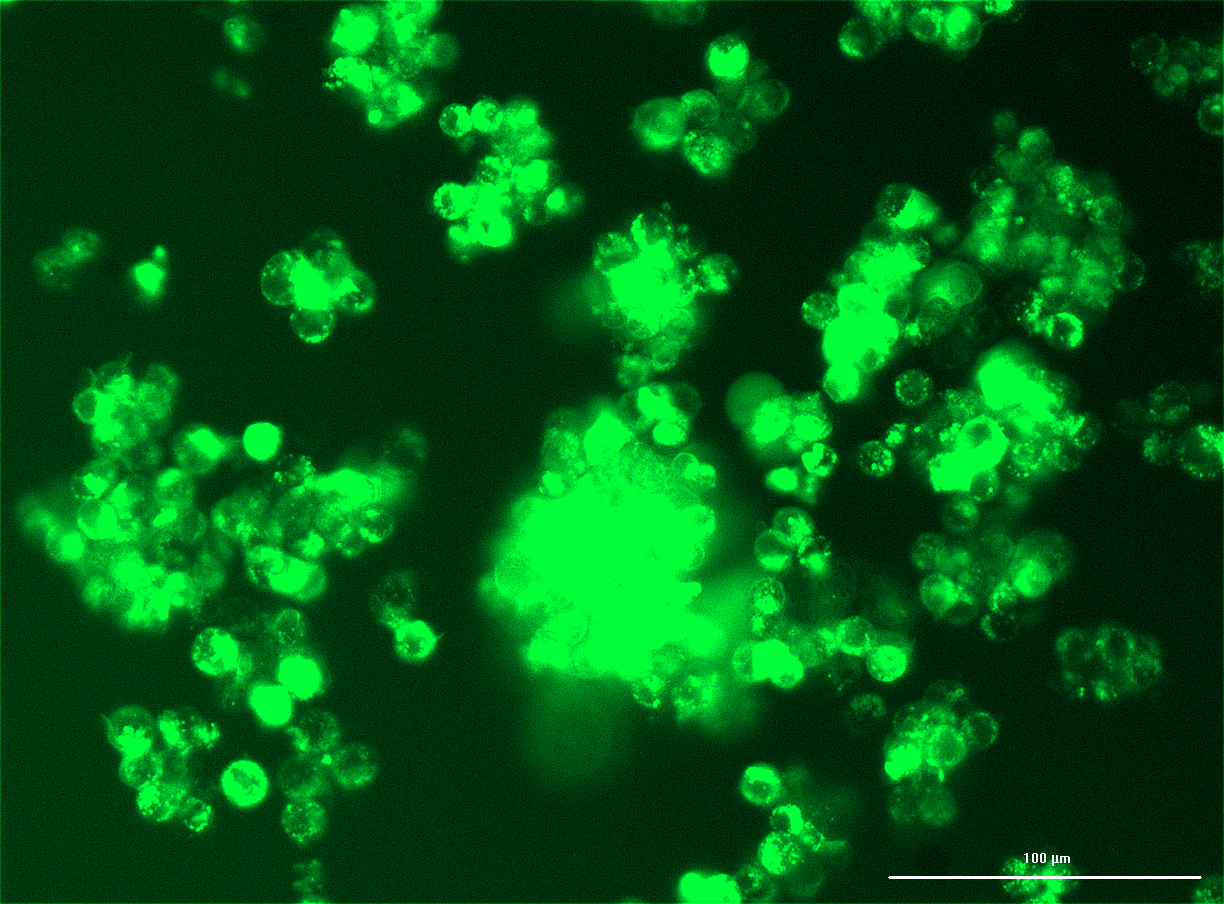

Supplement: Supplementary file 7 — Source data Fig. 2 [file 44321_2024_98_MOESM7_ESM.zip › Fig2/Fig2b/PD1.png]

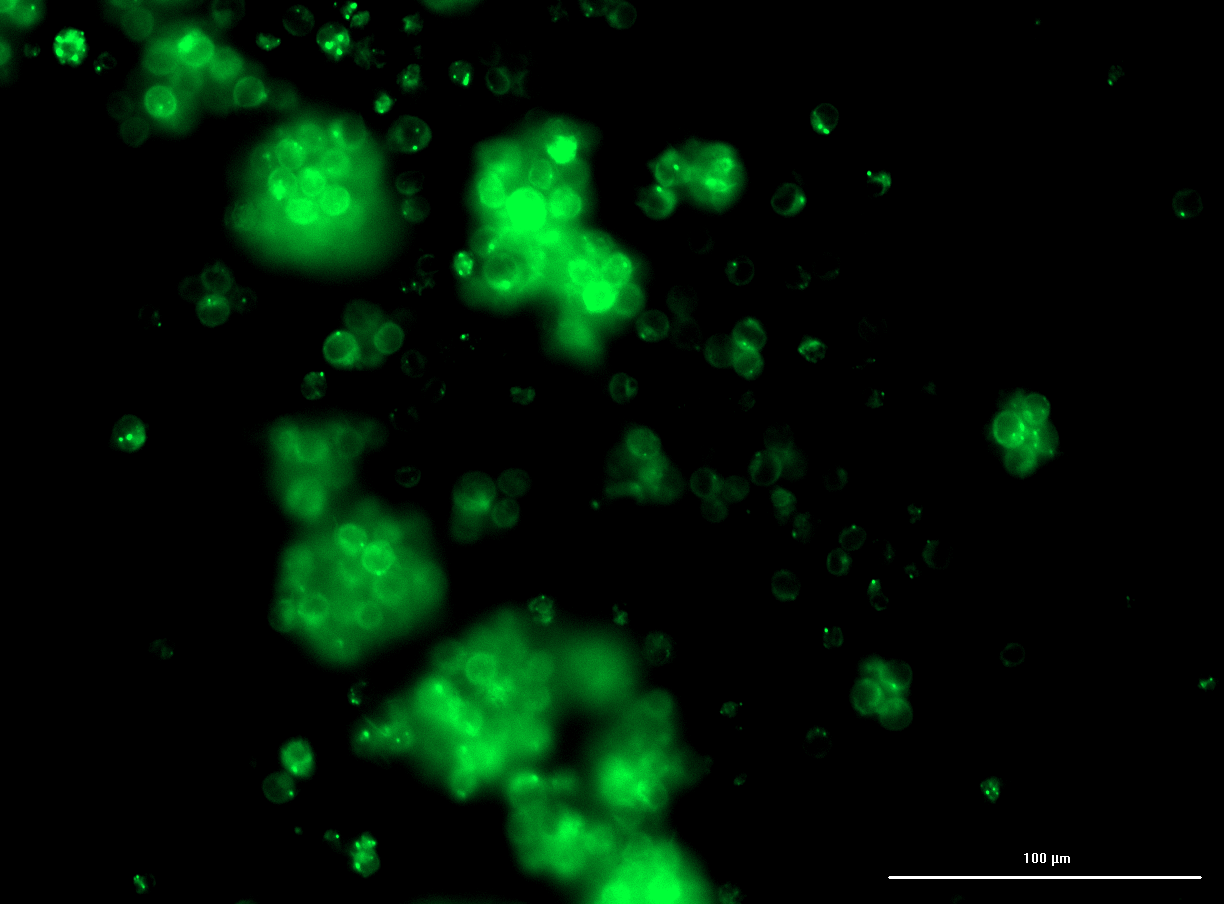

Supplement: Supplementary file 7 — Source data Fig. 2 [file 44321_2024_98_MOESM7_ESM.zip › Fig2/Fig2b/SC3.png]

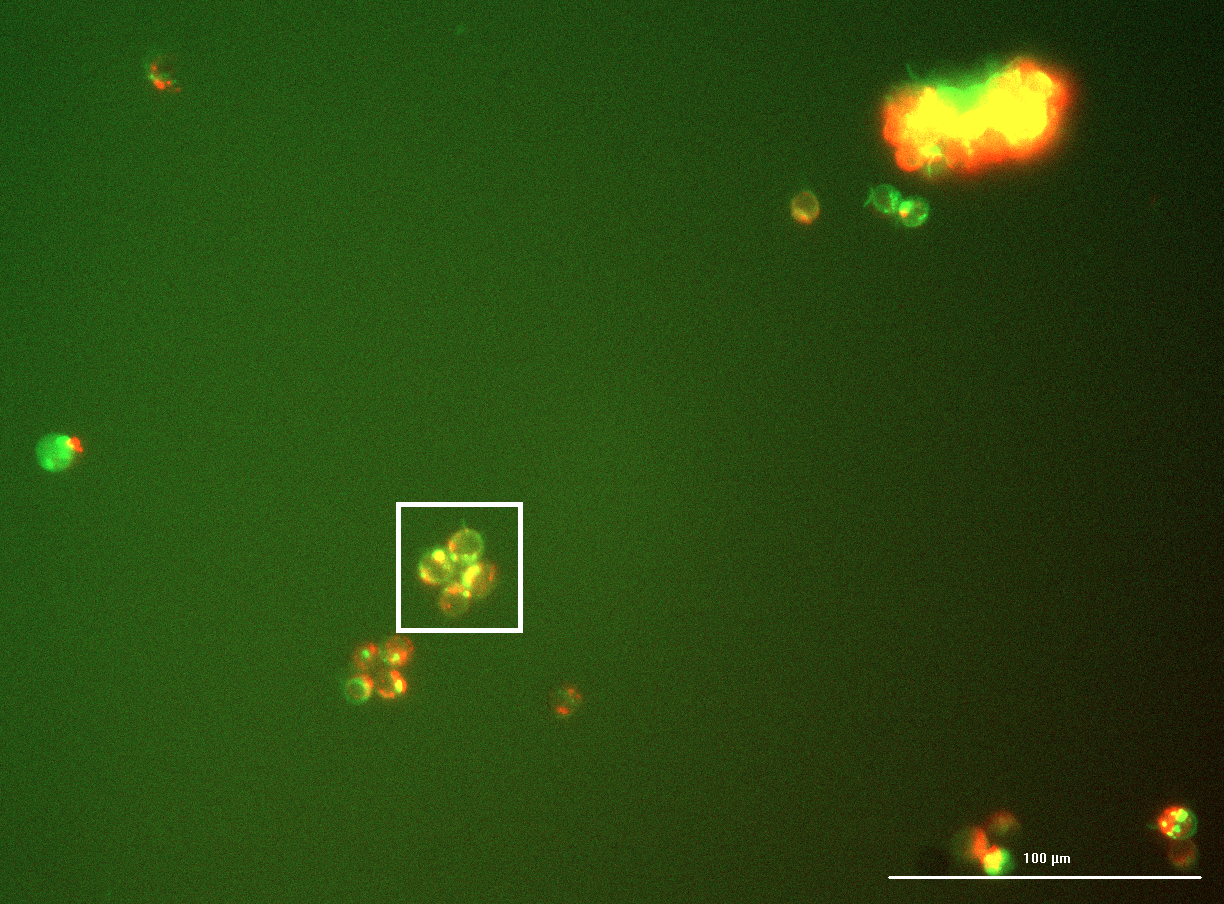

Supplement: Supplementary file 7 — Source data Fig. 2 [file 44321_2024_98_MOESM7_ESM.zip › Fig2/Fig2c /PD1+LAG3 with insert area.png]

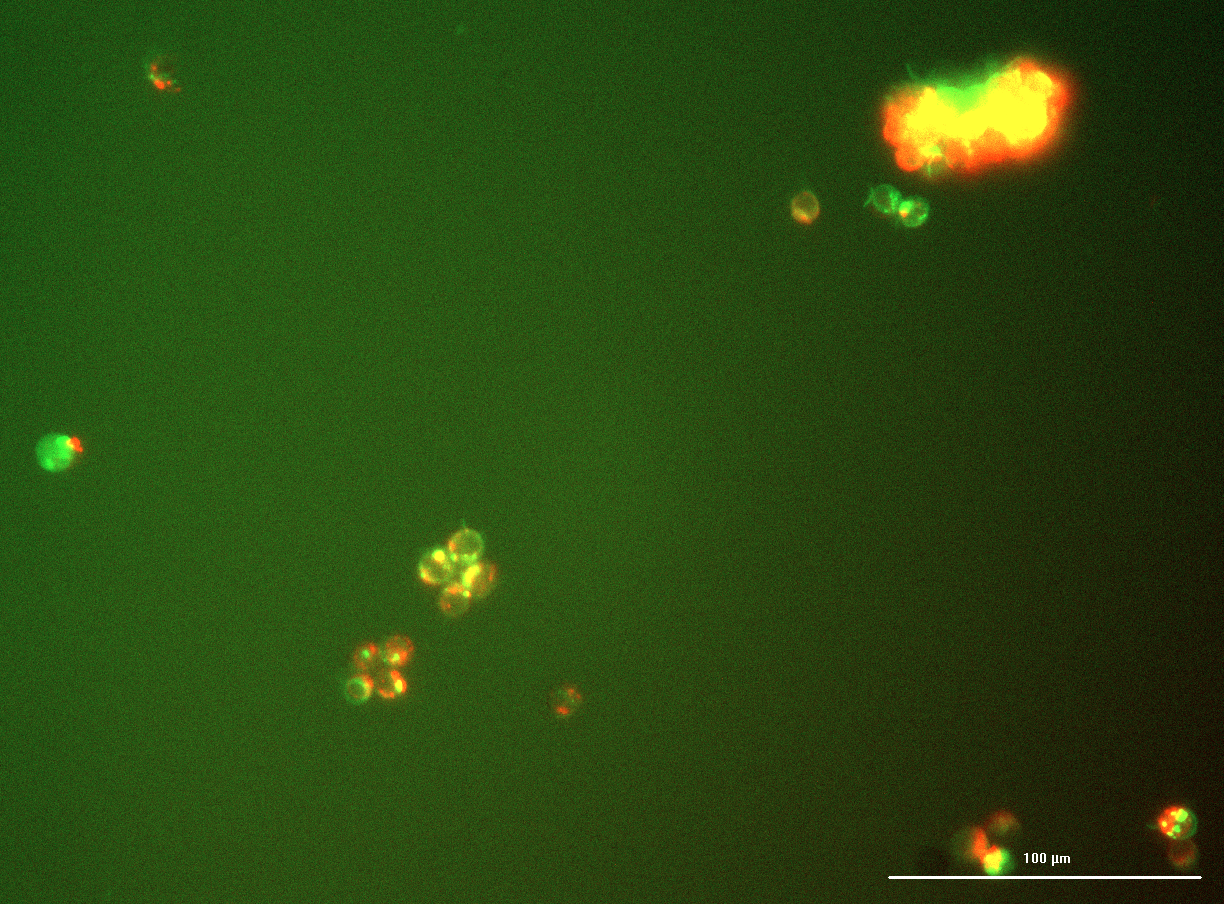

Supplement: Supplementary file 7 — Source data Fig. 2 [file 44321_2024_98_MOESM7_ESM.zip › Fig2/Fig2c /PD1+LAG3.png]

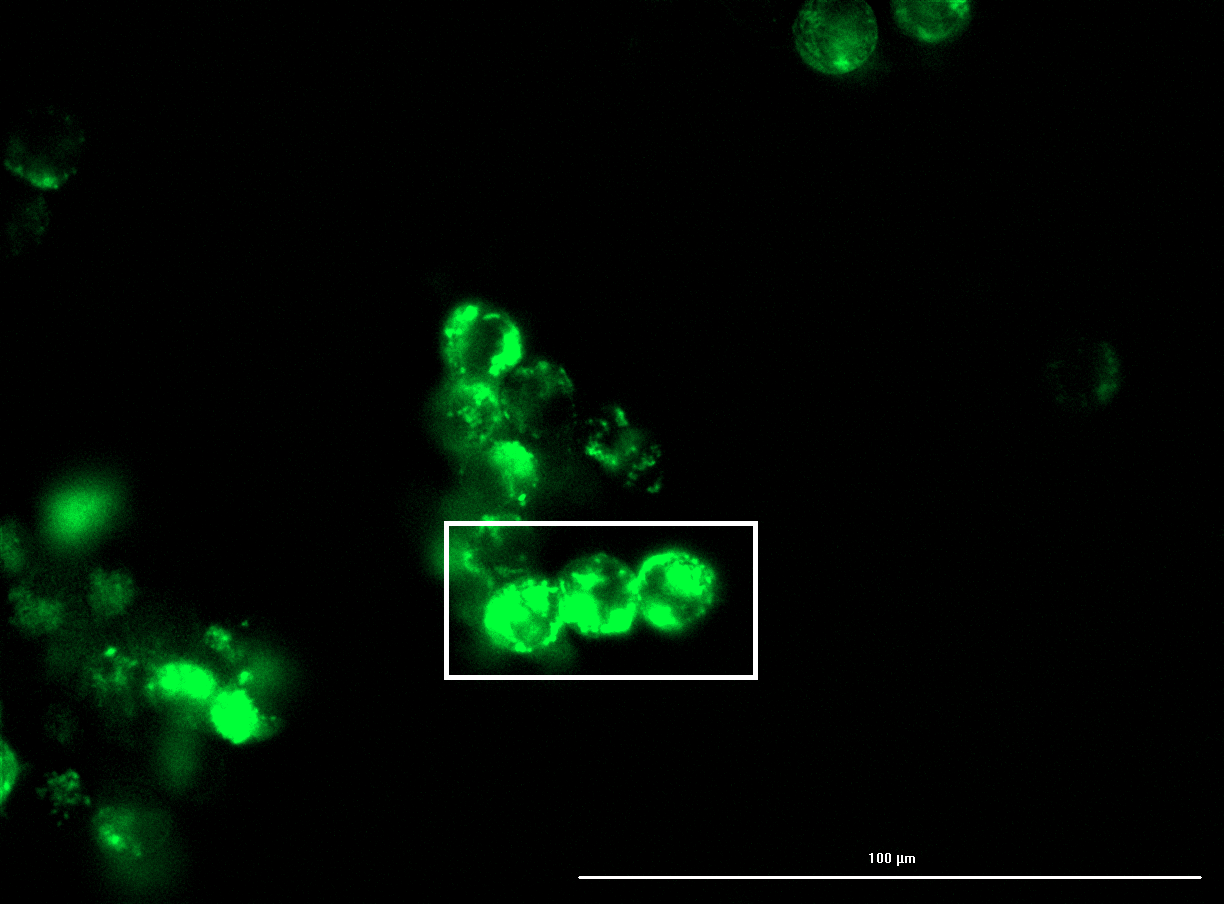

Supplement: Supplementary file 7 — Source data Fig. 2 [file 44321_2024_98_MOESM7_ESM.zip › Fig2/Fig2c /PD1 with insert area.png]

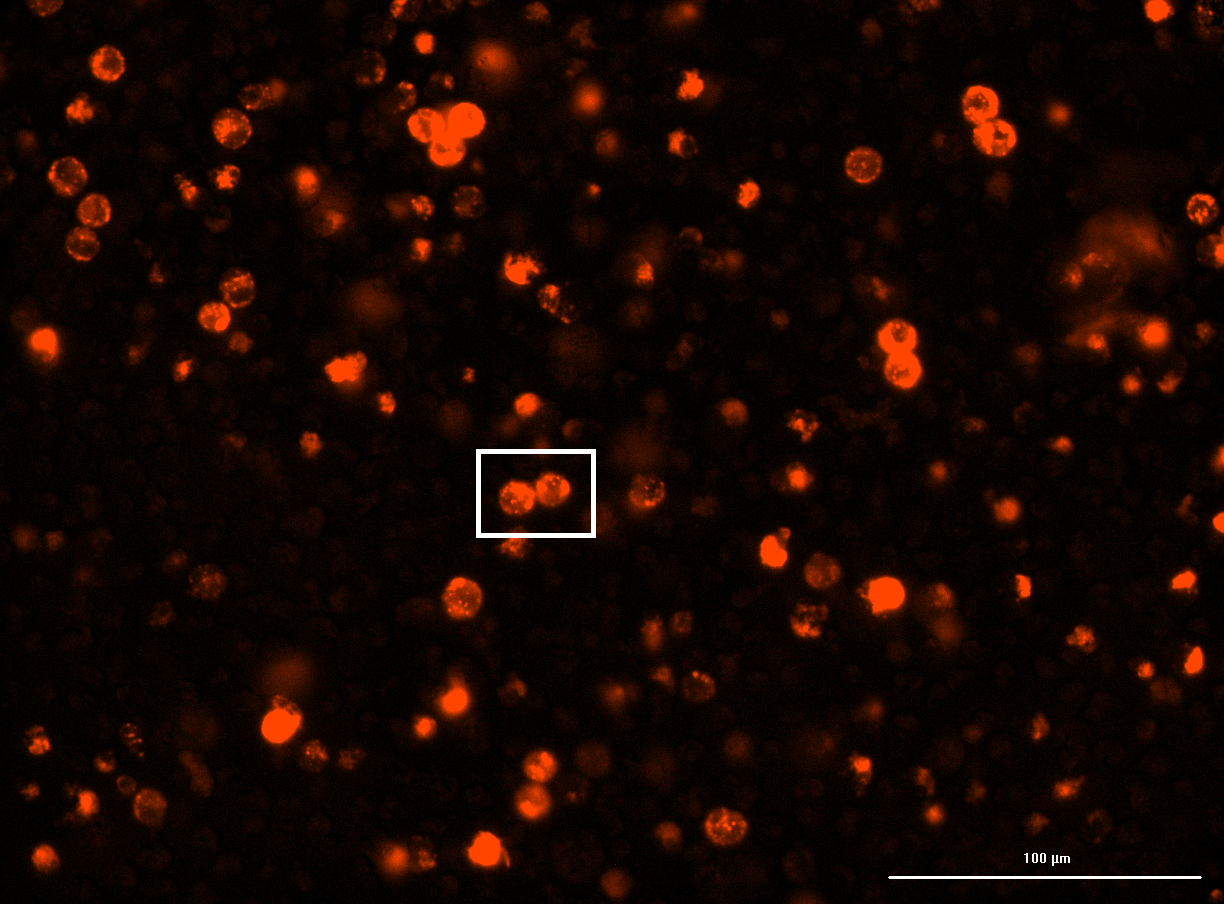

Supplement: Supplementary file 7 — Source data Fig. 2 [file 44321_2024_98_MOESM7_ESM.zip › Fig2/Fig2c /LAG-3 with insert area.png]

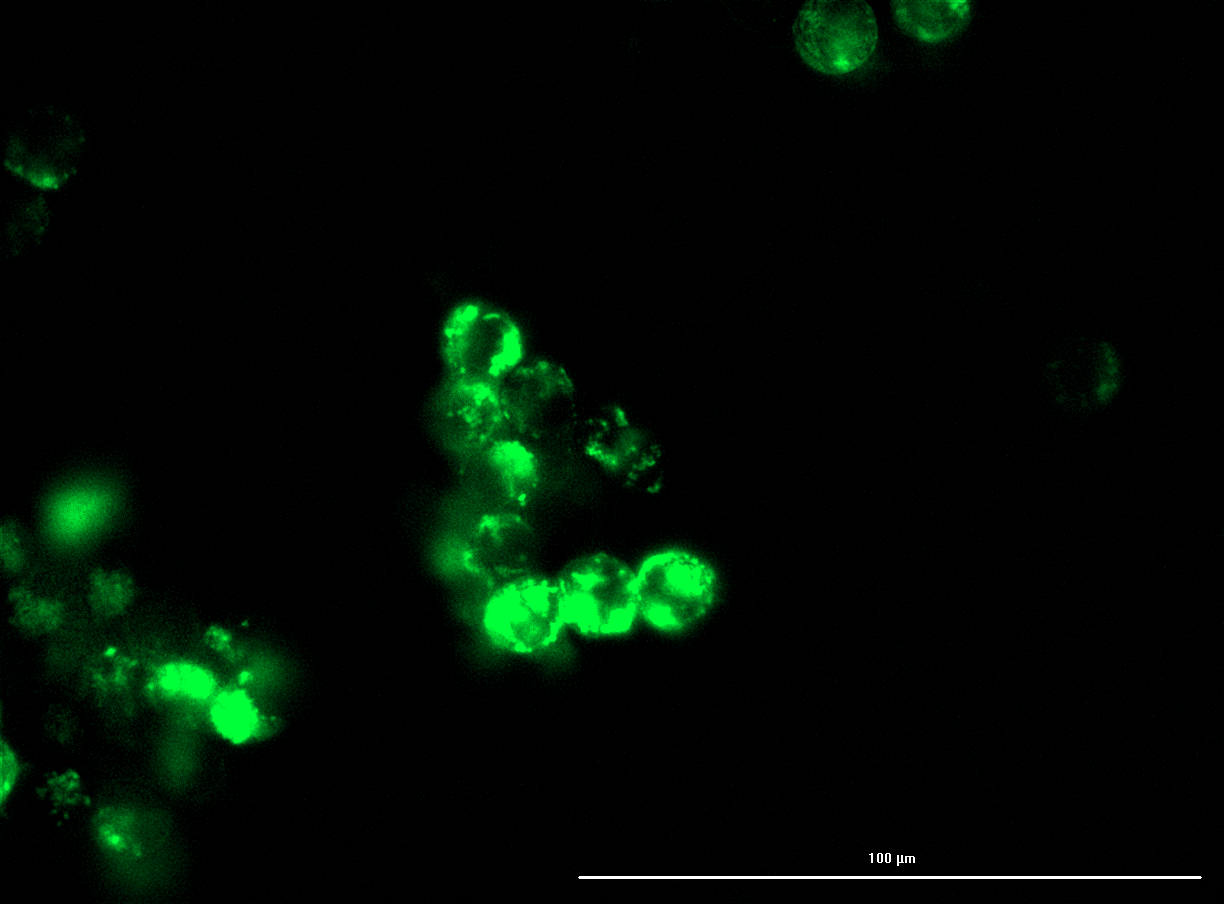

Supplement: Supplementary file 7 — Source data Fig. 2 [file 44321_2024_98_MOESM7_ESM.zip › Fig2/Fig2c /PD1.png]
