# Supplementary material for: PD-1/LAG-3 co-signaling profiling uncovers CBL ubiquitin ligases as key immunotherapy targets
Source: EMBO Mol Med. 2024 Jul 19;16(8):2. doi: 10.1038/s44321-024-00098-y (PMC11319776; doi:10.1038/s44321-024-00098-y)
Supplement: Supplementary file 14 — Expanded View Figures [file 44321_2024_98_MOESM14_ESM.pdf]

Expanded View Figures

Figure EV1. Correlation of *PDCD1* and *LAG3* expression with immune cell infiltrates.

(**A**) Heatmap of partial purity-adjusted Spearman's correlates calculated with TIMER 2.0. between *PDCD1/LAG3* expression and lymphoid infiltrates in a total number of 12159 samples distributed on TCGA cancers. (**B**) Heatmap of partial purity-adjusted Spearman's correlates calculated with TIMER 2.0. between *PDCD1/LAG3* expression and non-lymphoid infiltrates in a total number of 12159 samples distributed on TCGA cancers.

© The Author(s)

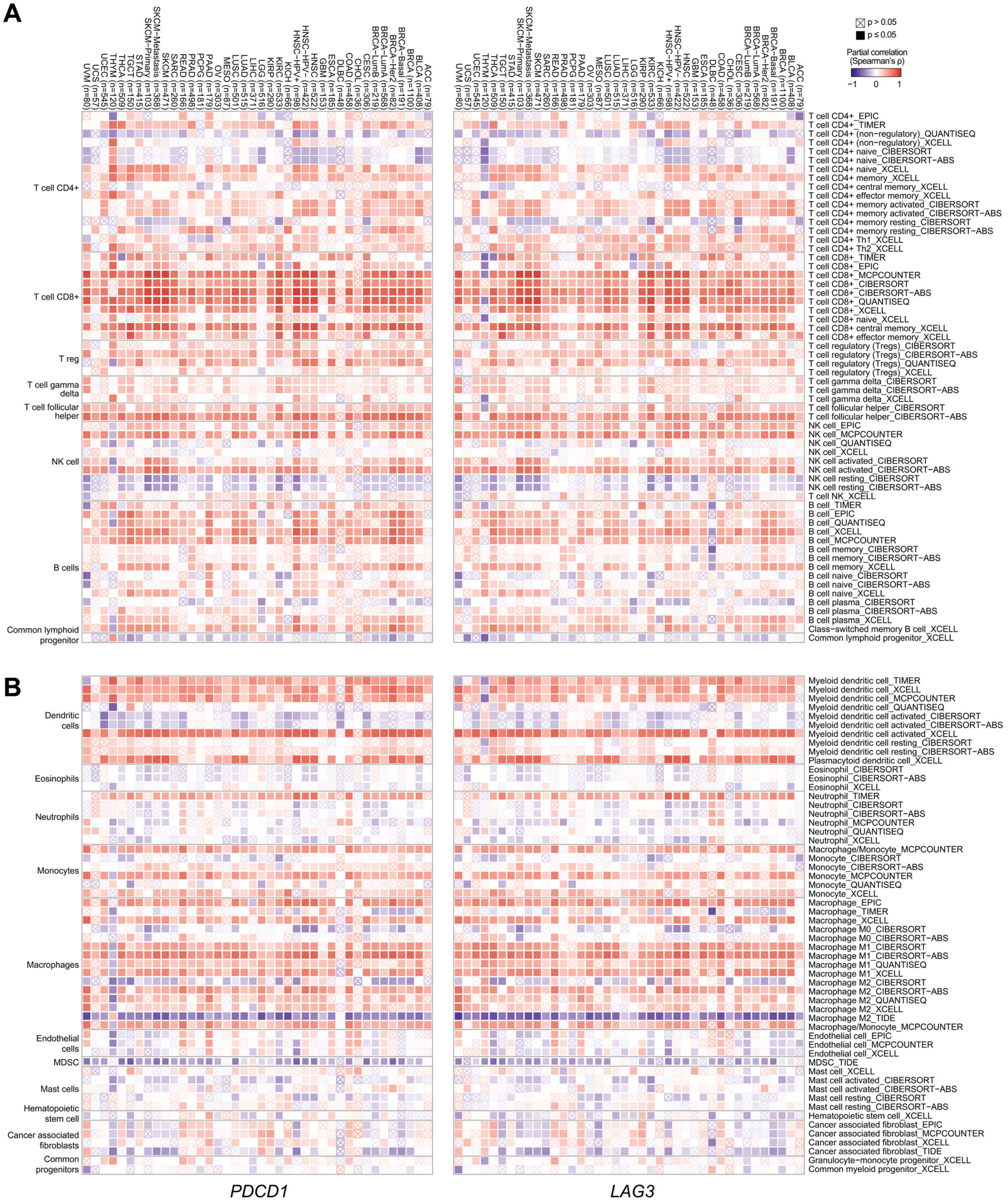
A
B
p > 0.05
p ≤ 0.05
Partial correlation
(Spearman's ρ)
−1
0
1
UVM (n=80)
UCS (n=57)
UCEC (n=545)
THYM (n=120)
THCA (n=509)
TGCT (n=150)
STAD (n=415)
SKCM−Metastasis (n=368)
SKCM−Primary (n=103)
SKCM (n=471)
SARC (n=260)
READ (n=166)
PRAD (n=498)
PCPG (n=181)
PAAD (n=179)
OV (n=303)
MESO (n=87)
LUSC (n=501)
LUAD (n=515)
LIHC (n=371)
LGG (n=516)
KIRP (n=290)
KIRC (n=533)
KICH (n=66)
HNSC−HPV+ (n=98)
HNSC−HPV− (n=422)
HNSC (n=522)
GBM (n=153)
ESCA (n=185)
DLBC (n=48)
COAD (n=458)
CHOL (n=36)
CESC (n=306)
BRCA−LumB (n=219)
BRCA−LumA (n=568)
BRCA−Her2 (n=82)
BRCA−Basal (n=191)
BRCA (n=1100)
BLCA (n=408)
ACC (n=79)
T cell CD4+
T cell CD8+
T reg
T cell gamma delta
T cell follicular helper
NK cell
B cells
Common lymphoid progenitor
T cell CD4+_EPIC
T cell CD4+_TIMER
T cell CD4+ (non−regulatory)_QUANTISEQ
T cell CD4+ (non−regulatory)_XCELL
T cell CD4+ naive_CIBERSORT
T cell CD4+ naive_CIBERSORT−ABS
T cell CD4+ naive_XCELL
T cell CD4+ memory_XCELL
T cell CD4+ central memory_XCELL
T cell CD4+ effector memory_XCELL
T cell CD4+ memory activated_CIBERSORT
T cell CD4+ memory activated_CIBERSORT−ABS
T cell CD4+ memory resting_CIBERSORT
T cell CD4+ memory resting_CIBERSORT−ABS
T cell CD4+ Th1_XCELL
T cell CD4+ Th2_XCELL
T cell CD8+_TIMER
T cell CD8+_EPIC
T cell CD8+_MCPCOUNTER
T cell CD8+_CIBERSORT
T cell CD8+_CIBERSORT−ABS
T cell CD8+_QUANTISEQ
T cell CD8+_XCELL
T cell CD8+ naive_XCELL
T cell CD8+ central memory_XCELL
T cell CD8+ effector memory_XCELL
T cell regulatory (Tregs)_CIBERSORT
T cell regulatory (Tregs)_CIBERSORT−ABS
T cell regulatory (Tregs)_QUANTISEQ
T cell regulatory (Tregs)_XCELL
T cell gamma delta_CIBERSORT
T cell gamma delta_CIBERSORT−ABS
T cell gamma delta_XCELL
T cell follicular helper_CIBERSORT
T cell follicular helper_CIBERSORT−ABS
NK cell_EPIC
NK cell_MCPCOUNTER
NK cell_QUANTISEQ
NK cell_XCELL
NK cell activated_CIBERSORT
NK cell activated_CIBERSORT−ABS
NK cell resting_CIBERSORT
NK cell resting_CIBERSORT−ABS
T cell NK_XCELL
B cell_TIMER
B cell_EPIC
B cell_QUANTISEQ
B cell_XCELL
B cell_MCPCOUNTER
B cell memory_CIBERSORT
B cell memory_CIBERSORT−ABS
B cell memory_XCELL
B cell naive_CIBERSORT
B cell naive_CIBERSORT−ABS
B cell naive_XCELL
B cell plasma_CIBERSORT
B cell plasma_CIBERSORT−ABS
B cell plasma_XCELL
Class−switched memory B cell_XCELL
Common lymphoid progenitor_XCELL
Dendritic cells
Eosinophils
Neutrophils
Monocytes
Macrophages
Endothelial cells
MDSC
Mast cells
Hematopoietic stem cell
Cancer associated fibroblasts
Common progenitors
Myeloid dendritic cell_TIMER
Myeloid dendritic cell_XCELL
Myeloid dendritic cell_MCPCOUNTER
Myeloid dendritic cell_QUANTISEQ
Myeloid dendritic cell activated_CIBERSORT
Myeloid dendritic cell activated_CIBERSORT−ABS
Myeloid dendritic cell activated_XCELL
Myeloid dendritic cell resting_CIBERSORT
Myeloid dendritic cell resting_CIBERSORT−ABS
Plasmacytoid dendritic cell_XCELL
Eosinophil_CIBERSORT
Eosinophil_CIBERSORT−ABS
Eosinophil_XCELL
Neutrophil_TIMER
Neutrophil_CIBERSORT
Neutrophil_CIBERSORT−ABS
Neutrophil_MCPCOUNTER
Neutrophil_QUANTISEQ
Neutrophil_XCELL
Macrophage/Monocyte_MCPCOUNTER
Monocyte_CIBERSORT
Monocyte_CIBERSORT−ABS
Monocyte_MCPCOUNTER
Monocyte_QUANTISEQ
Monocyte_XCELL
Macrophage_EPIC
Macrophage_TIMER
Macrophage_XCELL
Macrophage M0_CIBERSORT
Macrophage M0_CIBERSORT−ABS
Macrophage M1_CIBERSORT
Macrophage M1_CIBERSORT−ABS
Macrophage M1_QUANTISEQ
Macrophage M1_XCELL
Macrophage M2_CIBERSORT
Macrophage M2_CIBERSORT−ABS
Macrophage M2_QUANTISEQ
Macrophage M2_XCELL
Macrophage M2_TIDE
Macrophage/Monocyte_MCPCOUNTER
Endothelial cell_EPIC
Endothelial cell_MCPCOUNTER
Endothelial cell_XCELL
MDSC_TIDE
Mast cell_XCELL
Mast cell activated_CIBERSORT
Mast cell activated_CIBERSORT−ABS
Mast cell resting_CIBERSORT
Mast cell resting_CIBERSORT−ABS
Hematopoietic stem cell_XCELL
Cancer associated fibroblast_EPIC
Cancer associated fibroblast_MCPCOUNTER
Cancer associated fibroblast_XCELL
Cancer associated fibroblast_TIDE
Granulocyte−monocyte progenitor_XCELL
Common myeloid progenitor_XCELL
PDCD1
LAG3

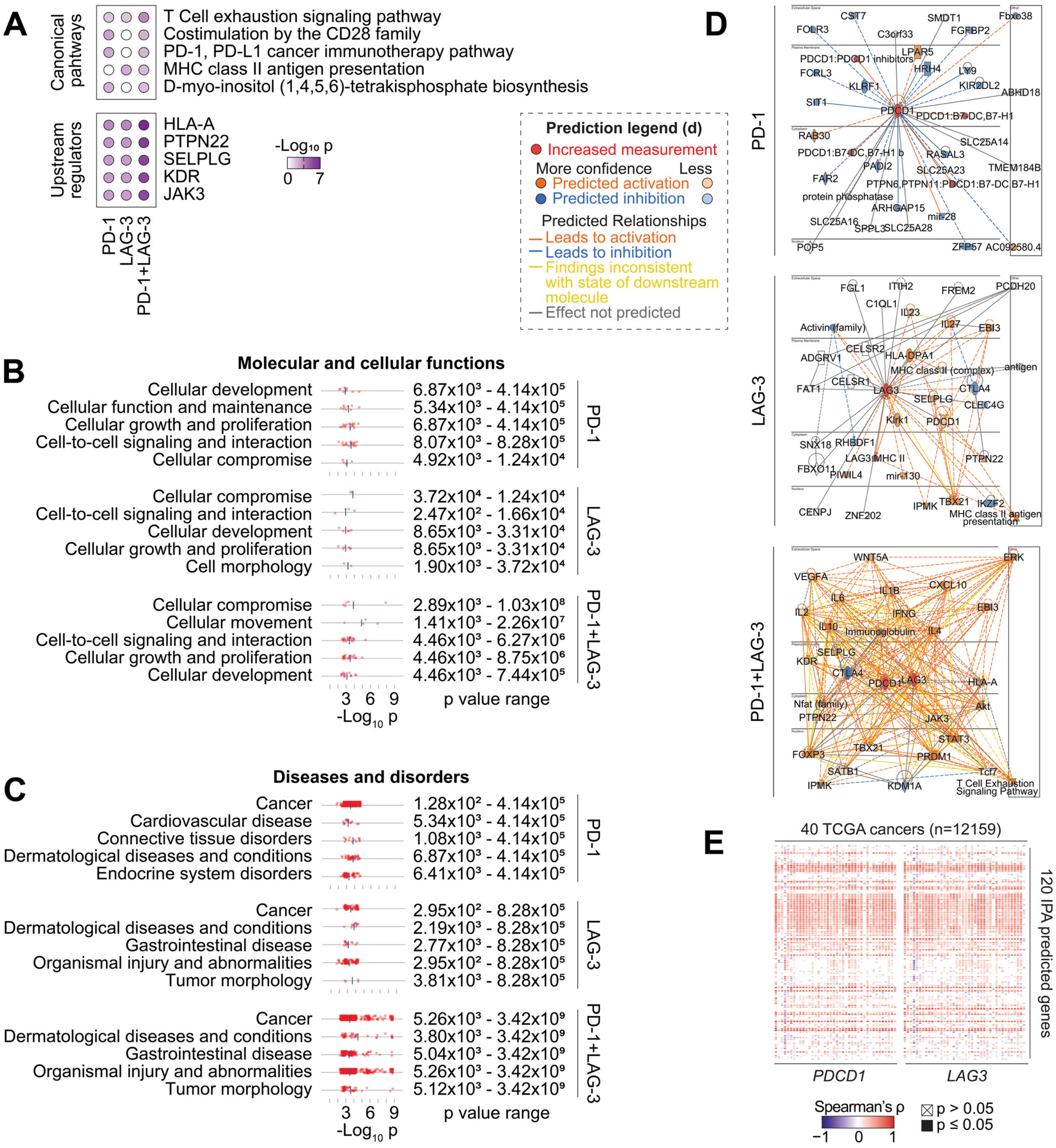
A
Canonical pathways
T Cell exhaustion signaling pathway
Costimulation by the CD28 family
PD-1, PD-L1 cancer immunotherapy pathway
MHC class II antigen presentation
D-myo-inositol (1,4,5,6)-tetrakisphosphate biosynthesis
Upstream regulators
HLA-A
PTPN22
SELPLG
KDR
JAK3
-Log10 p
0 7
PD-1
LAG-3
PD-1+LAG-3
Prediction legend (d)
Increased measurement
More confidence Less
Predicted activation
Predicted inhibition
Predicted Relationships
Leads to activation
Leads to inhibition
Findings inconsistent with state of downstream molecule
Effect not predicted
B
Molecular and cellular functions
Cellular development 6.87x10³ - 4.14x10⁵
Cellular function and maintenance 5.34x10³ - 4.14x10⁵
Cellular growth and proliferation 6.87x10³ - 4.14x10⁵
Cell-to-cell signaling and interaction 8.07x10³ - 8.28x10⁵
Cellular compromise 4.92x10³ - 1.24x10⁴
PD-1
Cellular compromise 3.72x10⁴ - 1.24x10⁴
Cell-to-cell signaling and interaction 2.47x10² - 1.66x10⁴
Cellular development 8.65x10³ - 3.31x10⁴
Cellular growth and proliferation 8.65x10³ - 3.31x10⁴
Cell morphology 1.90x10³ - 3.72x10⁴
LAG-3
Cellular compromise 2.89x10³ - 1.03x10⁸
Cellular movement 1.41x10³ - 2.26x10⁷
Cell-to-cell signaling and interaction 4.46x10³ - 6.27x10⁶
Cellular growth and proliferation 4.46x10³ - 8.75x10⁶
Cellular development 4.46x10³ - 7.44x10⁵
PD-1+LAG-3
3 6 9
-Log10 p
p value range
C
Diseases and disorders
Cancer 1.28x10² - 4.14x10⁵
Cardiovascular disease 5.34x10³ - 4.14x10⁵
Connective tissue disorders 1.08x10³ - 4.14x10⁵
Dermatological diseases and conditions 6.87x10³ - 4.14x10⁵
Endocrine system disorders 6.41x10³ - 4.14x10⁵
PD-1
Cancer 2.95x10² - 8.28x10⁵
Dermatological diseases and conditions 2.19x10³ - 8.28x10⁵
Gastrointestinal disease 2.77x10³ - 8.28x10⁵
Organismal injury and abnormalities 2.95x10² - 8.28x10⁵
Tumor morphology 3.81x10³ - 8.28x10⁵
LAG-3
Cancer 5.26x10³ - 3.42x10⁹
Dermatological diseases and conditions 3.80x10³ - 3.42x10⁹
Gastrointestinal disease 5.04x10³ - 3.42x10⁹
Organismal injury and abnormalities 5.26x10³ - 3.42x10⁹
Tumor morphology 5.12x10³ - 3.42x10⁹
PD-1+LAG-3
3 6 9
-Log10 p
p value range
D
PD-1
LAG-3
PD-1+LAG-3
E
40 TCGA cancers (n=12159)
120 IPA predicted genes
PDCD1
LAG3
Spearman's ρ
−1 0 1
p > 0.05
p ≤ 0.05

 © The Author(s)

Figure EV2. Regulatory networks and causal relationships associated with PD-1/LAG-3 signature.

(**A**) Identified enriched canonical pathways and upstream regulators for the upregulation of PD-1/LAG-3 and combinations. (**B**) Identified enriched molecular and cellular functions for the upregulation of PD-1/LAG-3 and combinations. (**C**) Identified enriched diseases and disorders for the upregulation of PD-1/LAG-3 and combinations. (**D**) Predicted regulatory interactomes and associated networks with the indicated PD-1 and LAG-3 signatures. Key nodes are shown, and inter-nodal lines represent functional relationships between nodes. In red, upregulated input molecules as indicated (PD-1 and LAG-3). In blue, downregulated input molecules as indicated (PD-1 and LAG-3). Blue lines, predicted inhibition; orange lines, predicted activation; grey indicates a predicted relationship with a non-predicted effect, and yellow lines, predicted relationship findings inconsistent with the state of the downstream molecule. (**E**) Heatmap of partial purity-adjusted Spearman's correlates calculated with TIMER 2.0. between *PDCD1/LAG3* expression and a selection of genes regulating identified by IPA of a total number of 12159 samples distributed on the indicated TCGA cancers. Data information: For (**A–D**), QIAGEN IPA algorithms were used (accessed on 2024), applied on data from curated publicly available datasets of RNA-seq, small RNA-seq, metabolomics, proteomics, microarrays including miRNA and SNP, and small-scale experiments. IPA utilizes two scores for inference; *P*-values from a Fisher's exact test to obtain an enrichment score, and a Z-score to assess the match of observed and predicted regulation patterns.

© The Author(s)

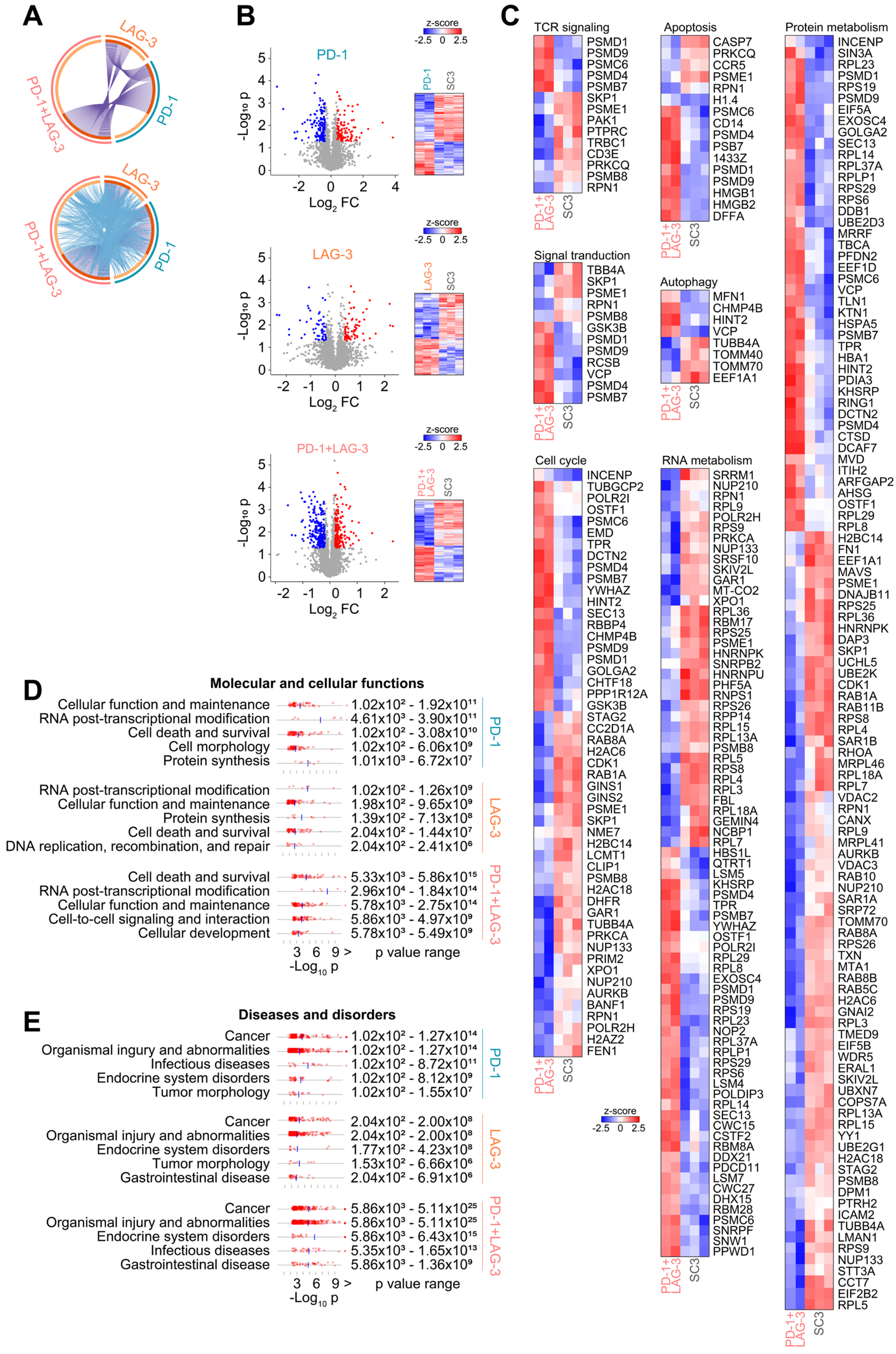
A
LAG-3
PD-1
PD-1+LAG-3
B
PD-1
LAG-3
PD-1+LAG-3
-Log10 p
Log2 FC
z-score
-2.5 0 2.5
SC3
C
TCR signaling
PSMD1 PSMD9 PSMC6 PSMD4 PSMB7 SKP1 PSME1 PAK1 PTPRC TRBC1 CD3E PRKCQ PSMB8 RPN1
Signal tranduction
TBB4A SKP1 PSME1 RPN1 PSMB8 GSK3B PSMD1 PSMD9 RCSB VCP PSMD4 PSMB7
Cell cycle
INCENP TUBGCP2 POLR2I OSTF1 PSMC6 EMD TPR DCTN2 PSMD4 PSMB7 YWHAZ HINT2 SEC13 RBBP4 CHMP4B PSMD9 PSMD1 GOLGA2 CHTF18 PPP1R12A GSK3B STAG2 CC2D1A RAB8A H2AC6 CDK1 RAB1A GINS1 GINS2 PSME1 SKP1 NME7 H2BC14 LCMT1 CLIP1 PSMB8 H2AC18 DHFR GAR1 TUBB4A PRKCA NUP133 PRIM2 XPO1 NUP210 AURKB BANF1 RPN1 POLR2H H2AZ2 FEN1
Apoptosis
CASP7 PRKCQ CCR5 PSME1 RPN1 H1.4 PSMC6 CD14 PSMD4 PSB7 1433Z PSMD1 PSMD9 HMGB1 HMGB2 DFFA
Autophagy
MFN1 CHMP4B HINT2 VCP TUBB4A TOMM40 TOMM70 EEF1A1
RNA metabolism
SRRM1 NUP210 RPN1 RPL9 POLR2H RPS9 PRKCA NUP133 SRSF10 SKIV2L GAR1 MT-CO2 XPO1 RPL36 RBM17 RPS25 PSME1 HNRNPK SNRPB2 HNRNPU PHF5A RNPS1 RPS26 RPP14 RPL15 RPL13A PSMB8 RPL5 RPS8 RPL4 RPL3 FBL RPL18A GEMIN4 NCBP1 RPL7 HBS1L QTRT1 LSM5 KHSRP PSMD4 TPR PSMB7 YWHAZ OSTF1 POLR2I RPL29 RPL8 EXOSC4 PSMD1 PSMD9 RPS19 RPL23 NOP2 RPL37A RPLP1 RPS29 RPS6 LSM4 POLDIP3 RPL14 SEC13 CWC15 CSTF2 RBM8A DDX21 PDCD11 LSM7 CWC27 DHX15 RBM28 PSMC6 SNRPF SNW1 PPWD1
Protein metabolism
INCENP SIN3A RPL23 PSMD1 RPS19 PSMD9 EIF5A EXOSC4 GOLGA2 SEC13 RPL14 RPL37A RPLP1 RPS29 RPS6 DDB1 UBE2D3 MRRF TBCA PFDN2 EEF1D PSMC6 VCP TLN1 KTN1 HSPA5 PSMB7 TPR HBA1 HINT2 PDIA3 KHSRP RING1 DCTN2 PSMD4 CTSD DCAF7 MVD ITIH2 ARFGAP2 AHSG OSTF1 RPL29 RPL8 H2BC14 FN1 EEF1A1 MAVS PSME1 DNAJB11 RPS25 RPL36 HNRNPK DAP3 SKP1 UCHL5 UBE2K CDK1 RAB1A RAB11B RPS8 RPL4 SAR1B RHOA MRPL46 RPL18A RPL7 VDAC2 RPN1 CANX RPL9 MRPL41 AURKB VDAC3 RAB10 NUP210 SAR1A SRP72 TOMM70 RAB8A RPS26 TXN MTA1 RAB8B RAB5C H2AC6 GNAI2 RPL3 TMED9 EIF5B WDR5 ERAL1 SKIV2L UBXN7 COPS7A RPL13A RPL15 YY1 UBE2G1 H2AC18 STAG2 PSMB8 DPM1 PTRH2 ICAM2 TUBB4A LMAN1 RPS9 NUP133 STT3A CCT7 EIF2B2 RPL5
PD-1+ LAG-3
SC3
z-score
-2.5 0 2.5
D
Molecular and cellular functions
Cellular function and maintenance 1.02x10² - 1.92x10¹¹
RNA post-transcriptional modification 4.61x10³ - 3.90x10¹¹
Cell death and survival 1.02x10² - 3.08x10¹⁰
Cell morphology 1.02x10² - 6.06x10⁹
Protein synthesis 1.01x10³ - 6.72x10⁷
PD-1
RNA post-transcriptional modification 1.02x10² - 1.26x10⁹
Cellular function and maintenance 1.98x10² - 9.65x10⁹
Protein synthesis 1.39x10² - 7.13x10⁸
Cell death and survival 2.04x10² - 1.44x10⁷
DNA replication, recombination, and repair 2.04x10² - 2.41x10⁶
LAG-3
Cell death and survival 5.33x10³ - 5.86x10¹⁵
RNA post-transcriptional modification 2.96x10⁴ - 1.84x10¹⁴
Cellular function and maintenance 5.78x10³ - 2.75x10¹⁴
Cell-to-cell signaling and interaction 5.86x10³ - 4.97x10⁹
Cellular development 5.78x10³ - 5.49x10⁹
PD-1+LAG-3
3 6 9 > p value range
-Log10 p
E
Diseases and disorders
Cancer 1.02x10² - 1.27x10¹⁴
Organismal ingury and abnormalities 1.02x10² - 1.27x10¹⁴
Infectious diseases 1.02x10² - 8.72x10¹¹
Endocrine system disorders 1.02x10² - 8.12x10⁹
Tumor morphology 1.02x10² - 1.55x10⁷
PD-1
Cancer 2.04x10² - 2.00x10⁸
Organismal injury and abnormalities 2.04x10² - 2.00x10⁸
Endocrine system disorders 1.77x10² - 4.23x10⁸
Tumor morphology 1.53x10² - 6.66x10⁶
Gastrointestinal disease 2.04x10² - 6.91x10⁶
LAG-3
Cancer 5.86x10³ - 5.11x10²⁵
Organismal injury and abnormalities 5.86x10³ - 5.11x10²⁵
Endocrine system disorders 5.86x10³ - 6.43x10¹⁵
Infectious diseases 5.35x10³ - 1.65x10¹³
Gastrointestinal disease 5.86x10³ - 1.36x10⁹
PD-1+LAG-3
3 6 9 > p value range
-Log10 p

 © The Author(s)

Figure EV3. Proteomes of T-cells with PD-1/LAG-3-regulated pathways.

(**A**) Circo plot representing the overlap from the input proteome dataset lists. Upper circle: On the outside, each arc represents the identity of each proteome. On the inside, each arc represents a list, where each gene has a spot on the arc. Dark orange color represents the proteins that appear in multiple lists and light orange color represents proteins that are unique to that list. Purple lines link the same protein that are shared by multiple lists. Above circle: On the outside, same as upper circle. On the inside, each arc represents a list, where each gene has a spot on the arc. Dark orange color represents the molecules that appear in multiple lists and light orange color represents molecules that are unique to that list. Purple lines link the same gene that are shared by multiple lists. Blue lines link the different genes where they fall into the same ontology term (the term has to statistically significantly enriched and with size no larger than 100). Blue links indicate the degree of functional overlap among the input lists. (**B**) Volcano plots and heatmap with the number of differentially regulated proteins in PD-1, LAG-3, and PD-1 + LAG-3 Jurkat T-cell lines compared to SC3 control cells (p-value ≤ 0.05) for upregulated (Red, Log_2 (Fold Change) ≥ 0.38) and downregulated (Blue, Log_2 (Fold Change) ≤ -0.38) proteins. Blue: significantly downregulated, red: significantly downregulated. Grey: not significantly regulated. (**C**) Heatmaps of differential protein expression in the PD-1 + LAG-3 proteomic dataset compared with the proteome of the SC3-Jurkat control cell line. Red, significantly upregulated proteins (p-value ≤ 0.05, Log_2 (Fold Change) ≥ 0.38); blue, significantly downmodulated proteins (p-value ≤ 0.05, Log_2 (Fold Change) ≤ -0.38). Relevant T-cell pathways and functions are indicated on top. Specific targets are indicated on the right. (**D**) Identified enriched molecular and cellular functions for the PD-1/LAG-3 proteomes. (**E**) Identified enriched diseases and disorders for the PD-1/LAG-3 proteomes. Data information: Statistical comparisons are shown in the graph as indicated in Methods. For (**B**, **C**), Perseus was used for statistical analyses. An unpaired Student t-test was used for direct comparisons between two groups of samples. Differential PD-1/LAG-3 proteins versus the SC3 control condition comparisons were identified, following p-value ≤ 0.05, Log_2 (Fold Change) ≥ 0.38 and Log_2 (Fold Change) ≤ -0.38 criteria. For (**D**, **E**), QIAGEN IPA algorithms were used (accessed on 2024), applied on data from curated publicly available datasets of RNA-seq, small RNA-seq, metabolomics, proteomics, microarrays including miRNA and SNP, and small-scale experiments. IPA utilizes two scores for inference; P-values from a Fisher's exact test to obtain an enrichment score, and a Z-score to assess the match of observed and predicted regulation patterns", as indicated in the rest of the legends. It is lacking the last part of the sentence by mistake, sorry for the inconvenience. Source data are available online for this figure.

© The Author(s)

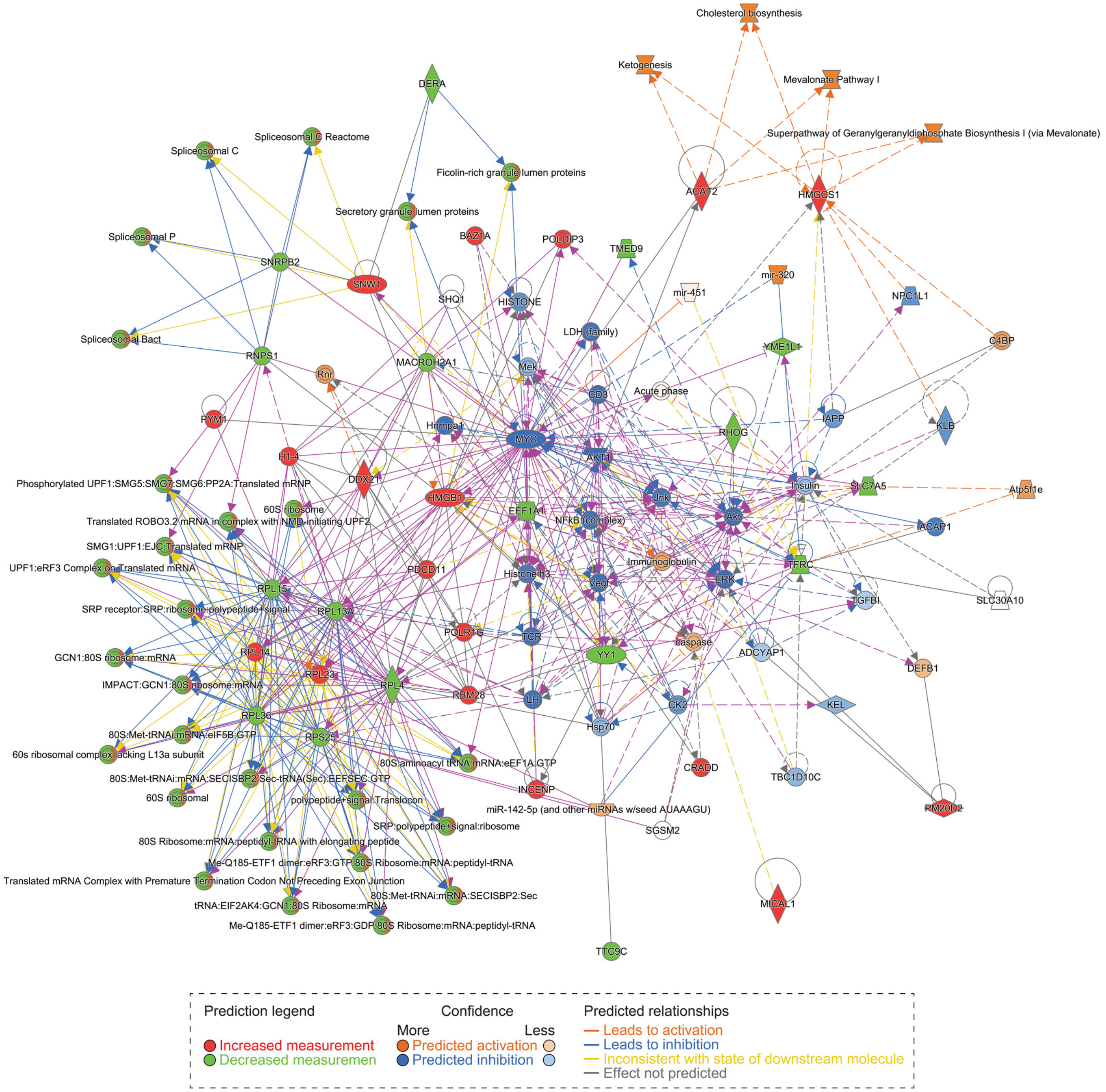

Figure EV4. Proteomic interactome networks associated to constitutive activation of PD-1/LAG-3 in Jurkat T-cell lines, generated by IPA.

Top networks describing potential molecular interactions of the 35 commonly regulated dataset molecules associated to the 35 commonly regulated proteins. In red, upregulated proteins. In green, downregulated proteins. Blue lines, predicted inhibition; orange lines, predicted activation; grey indicates a predicted relationship with a non-predicted effect, and yellow lines, predicted relationship findings inconsistent with the state of the downstream molecule. The specific legends to inter-nodal relationships are described in IPA (Ingenuity Pathway Analysis | QIAGEN Digital Insights). QIAGEN IPA algorithms were used (accessed on 2024), applied on data from curated publicly available datasets of RNA-seq, small RNA-seq, metabolomics, proteomics, microarrays including miRNA and SNP, and small-scale experiments. IPA utilizes two scores for inference; *P*-values from a Fisher's exact test to obtain an enrichment score, and a Z-score to assess the match of observed and predicted regulation patterns.

 © The Author(s)
